# The longitudinal association of remnant cholesterol with cardiovascular outcomes in patients with diabetes and pre-diabetes

**DOI:** 10.1186/s12933-020-01076-7

**Published:** 2020-07-06

**Authors:** Ye-Xuan Cao, Hui-Wen Zhang, Jing-Lu Jin, Hui-Hui Liu, Yan Zhang, Ying Gao, Yuan-Lin Guo, Na-Qiong Wu, Qi Hua, Yan-Fang Li, Xiao-Lin Li, Rui-Xia Xu, Chuan-Jue Cui, Geng Liu, Qian Dong, Jing Sun, Cheng-Gang Zhu, Jian-Jun Li

**Affiliations:** 1 State Key Laboratory of Cardiovascular Disease, Fu Wai Hospital, National Center for Cardiovascular Diseases, Chinese Academy of Medical Sciences, Peking Union Medical College, BeiLiShi Road 167, Beijing, 100037 China; 2 Department of Cardiology, Xuanwu Hospital, Capital Medical University, Beijing, China; 3 Department of Cardiology, Beijing Anzhen Hospital, Capital Medical University, Beijing, China

**Keywords:** Remnant cholesterol, Pre-diabetes mellitus, Cardiovascular events

## Abstract

**Background:**

The atherogenicity of remnant cholesterol (RC) has been underlined by recent guidelines, which was linked to coronary artery disease (CAD), especially for patients with diabetes mellitus (DM). This study aimed to examine the prognostic value of plasma RC in the patients with CAD under different glucose metabolism status.

**Methods:**

Fasting plasma RC were directly calculated or measured in 4331 patients with CAD. Patients were followed for the occurrence of major adverse cardiovascular events (MACEs) and categorized according to both glucose metabolism status [DM, pre-DM, normoglycemia (NG)] and RC levels. Cox proportional hazards model was used to calculate hazard ratios (HRs) with 95% confidence intervals.

**Results:**

During a mean follow-up of 5.1 years, 541 (12.5%) MACEs occurred. The risk for MACEs was significantly higher in patients with elevated RC levels after adjustment for potential confounders. No significant difference in MACEs was observed between pre-DM and NG groups (p > 0.05). When stratified by combined status of glucose metabolism and RC, highest levels of calculated and measured RC were significant and independent predictors of developing MACEs in pre-DM (HR: 1.64 and 1.98; both p < 0.05) and DM (HR: 1.62 and 2.05; both p < 0.05). High RC levels were also positively associated with MACEs in patients with uncontrolled DM. .

**Conclusions:**

In this large-scale and long-term follow-up cohort study, data firstly demonstrated that higher RC levels were significantly associated with the worse prognosis in DM and pre-DM patients with CAD, suggesting that RC may be a target for patients with impaired glucose metabolism.

## Background

Dyslipidemia is a well-established causal factor for coronary artery disease (CAD), which has been verified by a number of epidemiological and genetic studies, especially in patients with diabetes mellitus (DM) [1, 2]. Lowering plasma low-density lipoprotein cholesterol (LDL-C) is a pivotal approach to prevent CAD, which has been highly recommended by current guidelines [3, 4]. Yet patients with a substantial reduction in LDL-C, they still have a considerable residual cardiovascular risk [5]. In recent years, emerging evidence revealed that remnant cholesterol (RC) might contribute to this residual risk [6, 7]. RC is the cholesterol content of triglyceride-rich lipoproteins (TRLs), which is composed of chylomicron remnants (CR), very low-density lipoprotein (VLDL), and intermediate density lipoprotein (IDL) [5]. Experimental studies have shown that RC is involved in the formation and progression of atherosclerosis by multiple mechanisms, like direct accumulation in the arterial wall and enhanced inflammatory response [5, 8]. Mendelian randomization studies and recent guidelines also reported a causal association between genetically elevated RC and CAD [2, 3, 9]. Despite some previous clinical studies have examined the association between RC and major adverse cardiovascular events (MACEs) in the primary and secondary preventions [10–12], data about the prognostic implications of RC and MACEs in CAD patients with different glucose metabolism status is currently lacking.

During past decades, a large number of epidemiological studies have shown that patients with DM and pre-DM are at increased risk for CAD [13, 14]. There are many putative mechanisms suggesting that dysglycemia, including elevated levels of TG and TRLs, is linked to the development of atherosclerosis [15–17]. Data has suggested that serum RC concentrations are elevated in patients with DM and can predict myocardial function and future coronary outcomes [15, 18]. In addition, evidence has indicated that patients with pre-DM have higher tendency to develop DM and also have higher RC than those with normoglycemia (NG) [19, 20]. Based on our prior studies, pre-DM alone did not increase cardiovascular risk but result in bad prognosis when combined with other metabolic factors including hypertension and hyperlipoproteinemia [21, 22]. Moreover, no optimal glycemic cutoff for risk of CAD in pre-DM patients is accessed, therefore non-glycemic risk factors should be taken into consideration for risk stratification [23]. Of note, available studies give no hint on the prognostic value of RC in CAD patients with pre-DM.

Although it was difficult to assay RC levels due to their heterogeneous properties in the past, a simple and reliable measurement has already been developed and verified [10, 12]. In this multi-center cohort study, we aimed to evaluate the combined effect of RC and different glucose metabolism status on the clinical outcomes in patients with stable CAD on optimal lipid-lowering therapy.

## Methods

### Study design and populations

As described in the flowchart (Additional file 1: Figure S1), from March 2011 to March 2017, 5028 patients hospitalized for acute chest pain and diagnosed as CAD by coronary angiography were consecutively enrolled from three medical centers. The exclusion criteria were as follows: missing detailed data, age < 18 years, heart failure, severe liver and/or renal insufficiency, thyroid dysfunction, systematic inflammatory disease, or malignant disease.

The study protocol was approved by national and local ethics committees. The study was undertaken in accordance with the Declaration of Helsinki. All study participants gave written consent.

### Baseline characteristics

All participants enrolled were underwent detailed clinical examination by experienced physicians and nurses. CAD was defined as the presence of coronary stenosis ≥ 50% at least one major artery segment assessed by two experienced physicians according to coronary angiography. Smokers were defined as subjects who consumed tobacco products within the past year. Body mass index (BMI) was calculated as weight (kg) divided by height (m) squared. Hypertension was defined as repeated blood pressure measurements ≥ 140/90 mmHg and/or taking antihypertensive medication. A diagnosis of DM was defined when 1 of 4 criteria was met: fasting plasma glucose (FPG) ≥ 7.0 mmol/L; 2-h plasma glucose of the oral glucose tolerance test (2 h-OGTT) ≥ 11.1 mmol/L; symptoms of hyperglycemia plus random plasma glucose ≥ 11.1 mmol/L; or use of antidiabetic medication or insulin injections. Pre-DM was considered in patients with FPG between 5.6 and 6.9 mmol/L, haemoglobin A1c (HbA1c) between 5.7% and 6.4% or 2 h-OGTT glucose between 7.8 and 11.0 mmol/L. Patients without DM or pre-DM were defined as NG [14].

### Laboratory assays and measurements

Blood samples of all patients were collected from cubital vein after fasting for at least 12 h upon admission and stored at − 80 °C until analysis. Concentrations of total cholesterol (TC), TG, LDL-C, high density lipoprotein cholesterol (HDL-C) and apolipoprotein B (apoB) were directly measured using an automatic biochemistry analyzer (Hitachi 7150, Tokyo, Japan). The non-HDL-C value was calculated as TC minus HDL-C. The concentrations of glucose were measured by enzymatic hexokinase method, while HbA1c was measured using Tosoh Automated Glycohemoglobin Analyser (HLC-723G8, Tokyo, Japan). Lipoprotein (a) [Lp(a)] levels were assayed by an immunoturbidimetry method as previously described [22]. Directly measured RC (MRC) was obtained using a two-step automated assay developed by Denka Seiken (Tokyo, Japan), measuring the cholesterol content in CR, VLDL, and IDL specifically, with the aid of enzymes and surfactants. The cholesterol in other lipoproteins was removed in the first step, and then the cholesterol in the remaining remnant lipoprotein particles were determined (for further details see Additional file 1: Methods) [10, 12]. Inter-assay coefficients of variation for the RC assays were 4.8%. Calculated RC (CRC) was defined as TC minus LDL-C minus HDL-C [7].

### Endpoint assignment

All patients received standard lipid-lowering therapy for at least 3 months after they were discharged from the hospital, which consisted of 10 mg/d rosuvastatin or equivalent intensive statins plus 10 mg/d ezetimibe. Patients were followed up at 6-month intervals through direct interview or telephone by trained nurses or physicians who were blinded to the clinical data. MACEs were defined as fatal and nonfatal myocardial infarction (MI), fatal and nonfatal ischemic stroke, unstable angina pectoris (UAP), coronary revascularization, and cardiovascular death. MI was confirmed when medical records showed diagnostic symptom patterns, electrocardiogram changes, and increases in cardiac enzyme concentrations. Ischemic stroke was defined as new-onset neurological symptoms lasting more than 24 h with diagnostic CT or MRI. UAP was defined according to rest angina or new-onset severe angina without troponin elevation but that required hospitalization or coronary revascularization. Revascularization was defined as percutaneous coronary intervention (PCI) or coronary artery bypass grafting (CABG) later than 90 days after discharged. Cardiovascular death was confirmed with information from hospital records, death certificates, and family contact. Thirty-six (0.7%) patients were lost to follow-up. Finally, a total of 4331 patients completed the follow-up and were included in the present study.

### Statistical analysis

The values were expressed as the number (percentage) for the categorical variables and the mean ± standard deviation (SD) or median (Q1-Q3 quartiles) for the continuous variables. Differences were assessed by Student’s *t*-test, the Mann–Whitney *U*-test, analysis of variance (ANOVA), χ^2^ analysis and Fisher’s test as appropriate. Pearson correlation coefficients were used to evaluate the relationship between plasma lipids and other biomarkers. Comparisons of Kaplan–Meier curves were performed with the Log-Rank test. Data with skewed distribution were logarithmically transformed before statistical analysis. Cox proportional hazards analysis with hazard ratios (HRs) and 95% confidence intervals (CIs) were used to evaluate the association of risk factors with MACEs in univariate and multivariate settings with backward elimination. Confounders included age, sex, BMI, smoking, hypertension, baseline statin, family history of CAD, TC, LDL-C, non-HDL-C, apo(B) and TG were entered into the multivariate model. Restricted cubic splines (RCS) adjusted for age and sex were created to assess linearity assumptions of the relationship between RC and MACEs. A p value < 0.05 was considered statistically significant. All statistical analyses were performed using SPSS version 24.0 software (SPSS Inc., Chicago, IL, USA).

## Results

### Baseline characteristics

The baseline characteristics of 4331 participants (mean age, 58.33 ± 12.29 years; men, 71.1%) are shown in Table 1. Among 4331 participants, 776 (17.9%), 1163 (26.9%), and 2392 (55.2%) were diagnosed as NG, pre-DM, and DM, respectively. The percentage of male patients was less in pre-DM and DM groups while the proportion of smoking patients was higher among individuals with impaired glucose metabolism (p for trend < 0.05). There was no significant difference regarding family history of CAD, hypertension, and baseline statin use among the three groups (p for trend > 0.05). The BMI, HbA1c, TC, and TG were positively associated with the status of glucose metabolism from NG to DM (all p for trend < 0.05). Patients with pre-DM and DM had higher levels of FPG compared with the NG group. The concentrations of CRC and MRC were elevated according to the status of glucose metabolism from NG to DM (both p for trend < 0.05). Additional file 1: Table S1 shows the correlation coefficients of RC and other risk factors. CRC and MRC showed moderate correlations with TG and TC. The distribution of CRC and MRC is shown in Additional file 1: Figure S2. When a linear regression was applied, MRC elevated 0.47 mmol/L per 1-mmol/L increase in CRC with *R*^2^ = 0.74.

**Table 1:** Baseline characteristics of study patients

Variables	Overall (N = 4331)	NG (N = 776)	Pre-DM (N = 1163)	DM (N = 2392)	p-value
Age, years	58.32 ± 12.29	54.93 ± 9.91	59.55 ± 17.36	58.82 ± 9.50	< 0.001
Male, n (%)	3078 (71.1)	594 (76.5)	802 (69.0)	1682 (70.3)	0.001
BMI, kg/(m^2^)	25.85 ± 3.10	25.34 ± 3.12	25.83 ± 3.10	26.23 ± 3.03	< 0.001
Family history of CAD, n (%)	608 (14.0)	128 (16.5)	152 (13.1)	328 (13.7)	0.082
Smoking, n (%)	2343 (54.1)	412 (53.1)	636 (54.7)	1317 (55.1)	0.003
Drinking, n (%)	1425 (32.9)	273 (35.2)	363 (31.2)	789 (33.0)	0.189
Hypertension, n (%)	2845 (65.7)	494 (63.7)	746 (64.1)	1605 (67.1)	0.058
FPG, mmol/L	6.37 ± 1.99	4.79 ± 0.42	6.62 ± 2.12	6.77 ± 1.93	0.006
HbA1C, %	6.67 ± 1.22	5.37 ± 0.23	6.02 ± 0.25	7.40 ± 1.31	0.002
TC, mmol/L	4.08 ± 1.05	4.01 ± 1.01	4.04 ± 1.04	4.11 ± 1.06	0.02
HDL-C, mmol/L	1.06 ± 0.29	1.07 ± 0.31	1.04 ± 0.28	1.06 ± 0.29	0.033
Non-HDL-C, mmol/L	2.89 (2.3–3.59)	2.78 (2.21–3.53)	2.88 (2.34–3.57)	2.92 (2.31–3.64)	0.013
LDL-C, mmol/L	2.44 ± 0.89	2.38 ± 0.87	2.43 ± 0.87	2.46 ± 0.91	0.081
TG, mmol/L	1.46 (1.09–2.02)	1.36 (0.99–1.89)	1.46 (1.12–2.02)	1.50 (1.11–2.06)	0.002
CRC, mmol/L	0.52 (0.36–0.73)	0.48 (0.34–0.68)	0.52 (0.36–0.73)	0.54 (0.37–0.75)	0.001
MRC, mmol/L	0.50 (0.36–0.69)	0.47 (0.34–0.66)	0.48 (0.36–0.67)	0.52 (0.37–0.71)	< 0.001
Lp(a), mg/dL	15.10 (6.72–36.12)	14.92 (6.91–35.71)	14.86 (6.42–34.04)	15.35 (6.87–37.35)	0.334
ApoA1, g/L	1.33 ± 0.29	1.32 ± 0.31	1.33 ± 0.28	1.33 ± 0.29	0.685
ApoB, g/L	0.91 ± 0.29	0.89 ± 0.28	0.91 ± 0.27	0.91 ± 0.30	0.356
HsCRP, mg/L	1.36 (0.74–2.87)	1.09 (0.62–2.36)	1.41 (0.77–2.96)	1.45 (0.77–3.06)	0.001
Baseline statin use, n (%)	2621 (60.5)	457 (58.9)	681 (58.6)	1483 (62.0)	0.085
Baseline ezetimibe use, n (%)	463 (10.7)	76 (9.8)	120 (10.3)	267 (11.2)	0.502
Follow-up statin, n (%)	4201 (97.0)	745 (96.0)	1132 (97.3)	2324 (97.2)	0.323
Antidiabetic drug					
OADs, n (%)	1468 (33.9)	–	–	1468 (61.4)	–
Insulin, n (%)	789 (18.2)	–	–	789 (33.0)	–

Data are expressed as the mean value ± SD, median with 25th and 75th or number (%)

NG, normoglycemia; DM, diabetes mellitus; BMI, body mass index; CAD, coronary artery disease; FPG, fasting plasma glucose; HbA1c, hemoglobin A1c; TC, total cholesterol; HDL-C, high-density lipoprotein cholesterol; LDL-C, low-density lipoprotein cholesterol; TG, triglyceride; CRC, calculated remnant cholesterol; MRC, measured remnant cholesterol; Lp(a), lipoprotein (a); ApoA1, apolipoprotein A 1; ApoB, apolipoprotein B; hsCRP, high sensitivity C-reactive protein; OADs, Oral antidiabetic agents

### Predictive role of RC in MACEs

Over a mean follow-up time of 5.1 years (IQR: 3.9–6.4), a total of 541 MACEs occurred, representing 26.7 (95% CI 17.8–38.1) events per 1000 person-years. Among patients with events, 75 (1.7%) died, 132 (3.0%) had UAP requiring hospitalization, 44 (1.0%) developed MI, 109 (2.5%) had stroke and 181 (4.2%) underwent post-discharge PCI or CABG.

**Fig. 1:**
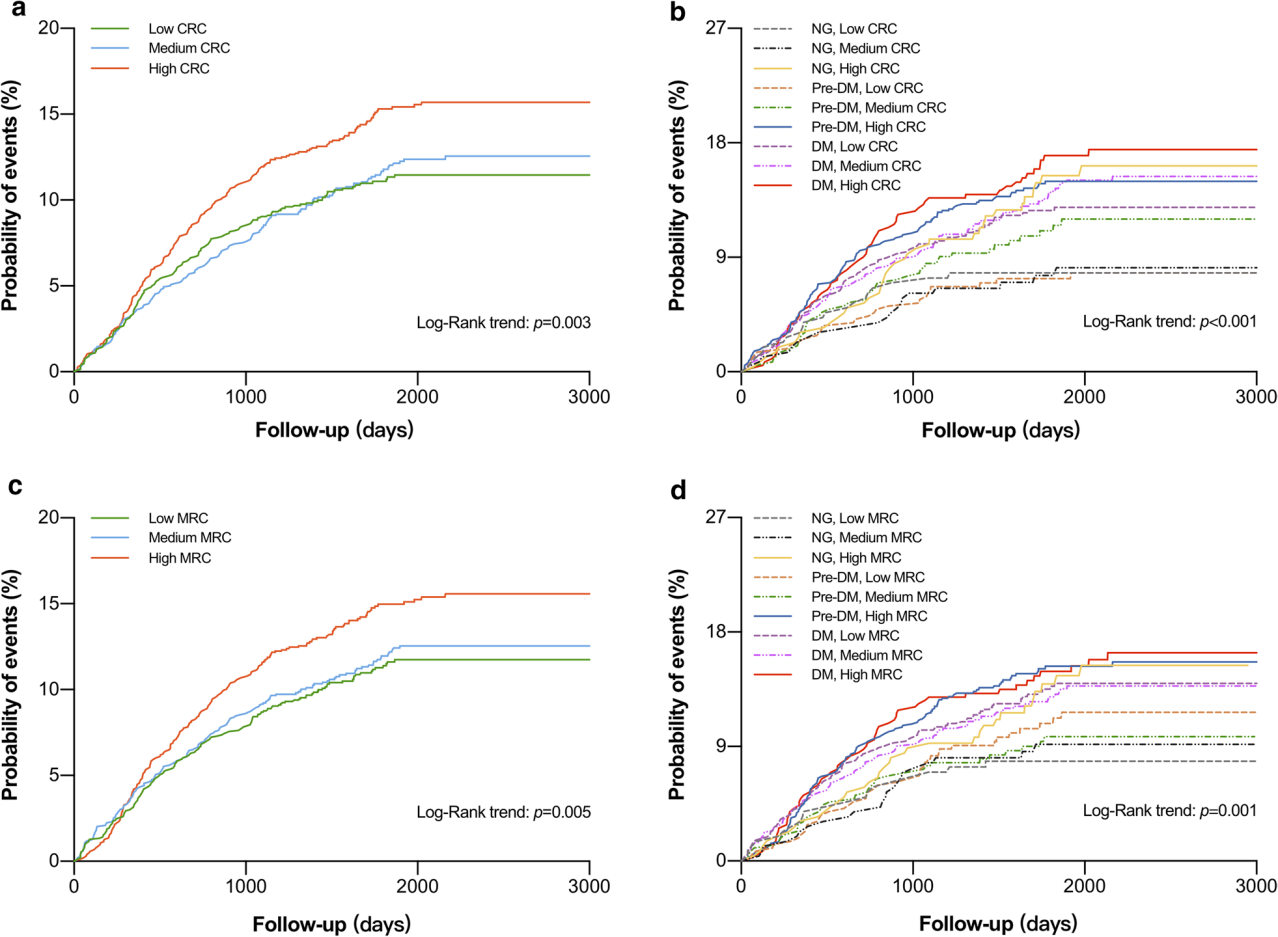
Kaplan-Meier analysis according to different glucose metabolism status and different remnant cholesterol levels. CRC, calculated remnant cholesterol; MRC, measured remnant cholesterol; NG, normoglycemia; DM, diabetes mellitus; MACE, major adverse cardiovascular event

Kaplan–Meier analysis demonstrated a significantly higher probability of developing MACEs in patients within the highest tertiles of CRC and MRC compared with those within the lowest tertiles (log-rank p < 0.01, Fig. 1). As shown in Additional file 1: Table S2, S3, multivariate Cox proportional hazard regression models revealed significant associations of plasma CRC and MRC per log-unit increase with incident MACEs (HR: 1.97, 95% CI 1.28–3.02, p = 0.002; HR: 1.54, 95% CI 1.27–1.86, p < 0.001). When analyzed as categorical variables, adjusted HRs for incident MACEs risk at the highest levels of the CRC and MRC compared with the lowest levels were 1.47 (95% CI 1.20–1.81) and 1.42 (95% CI 1.16–1.75) (Additional file 1: Table S4). As shown in Additional file 1: Figure S3, RCS showed strong trends toward positive associations between RC and MACEs.

### Glucose metabolism, RC and cardiovascular outcomes

The prevalence of MACEs in NG, pre-DM, and DM group was 10.1%, 11.7%, and 13.7%, respectively. Kaplan–Meier analysis (Additional file 1: Figure S4) showed that DM subjects had the lowest event-free survival rates among the 3 groups, while there was no significant difference between that of pre-DM and NG groups. As presented in Additional file 1: Table S4, multivariate Cox regression models showed that patients with DM had 1.35-fold higher risk of MACEs than NG subjects (HR: 1.35, 95% CI 1.06–1.73, p = 0.017), while patients with pre-DM did not show an increased risk in MACEs when compared with NG group (p > 0.05).

**Fig. 2:**
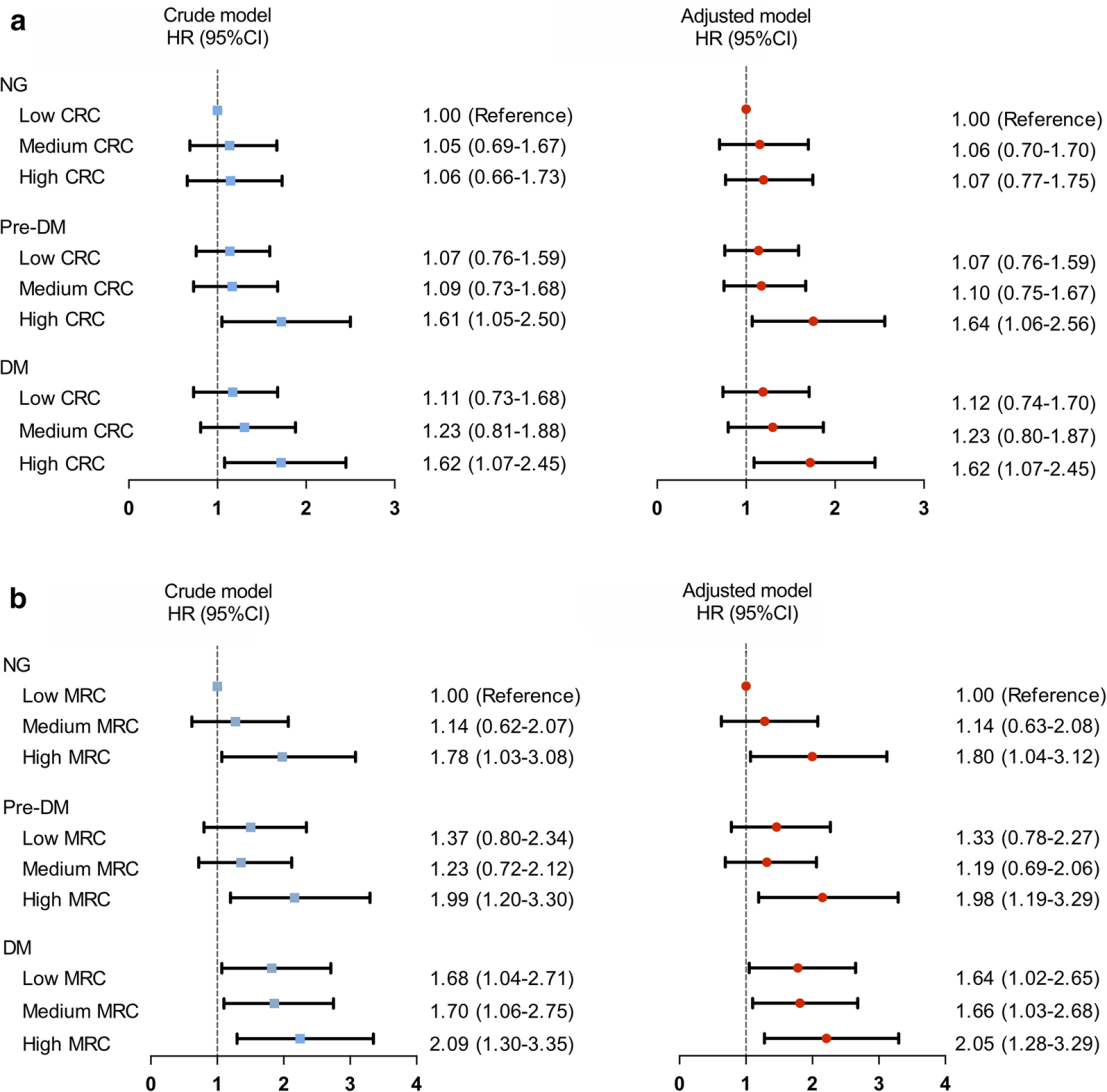
Remnant cholesterol levels in relation to cardiovascular events in patients with different glucose metabolism. HR, hazard ratio; CI, confidence interval; CRC, calculated remnant cholesterol; MRC, measured remnant cholesterol; NG, normoglycemia; DM, diabetes mellitus. Model adjusted for age, sex, body mass index, smoking, hypertension, baseline statin, family history of coronary artery disease, total cholesterol, low-density lipoprotein cholesterol, non-high lipoprotein cholesterol, apolipoprotein B and triglyceride

When the patients were evaluated according to both glucose metabolism and RC status (low, medium, and high for T1 to T3), Kaplan–Meier analysis showed that pre-DM plus high CRC and DM plus high CRC groups had significantly lower cumulative event-free survival rates compared with the reference group (NG plus low CRC; log-rank p < 0.001, Fig. 1). Similar results were observed in pre-DM plus high MRC and DM plus high MRC groups (log-rank p = 0.001). As shown in Fig. 2 and Additional file 1: Table S5, when both glucose metabolism and CRC status were incorporated as stratifying factors, multivariate Cox regression models indicated that patients in pre-DM plus high CRC and DM plus high CRC had higher risk of MACEs than patients in NG plus low CRC (HR: 1.64, 95% CI 1.06–2.56; HR: 1.62, 95% CI 1.07–2.45). Multivariate Cox regression analyses also found that high MRC plus pre-DM group and high MRC plus DM group were associated with 1.98- and 2.05-fold increased risk of MACEs (HR: 1.98, 95% CI 1.19–3.29; HR: 2.05, 95% CI 1.28–3.29).

### RC and MACEs in controlled and uncontrolled DM

**Fig. 3:**
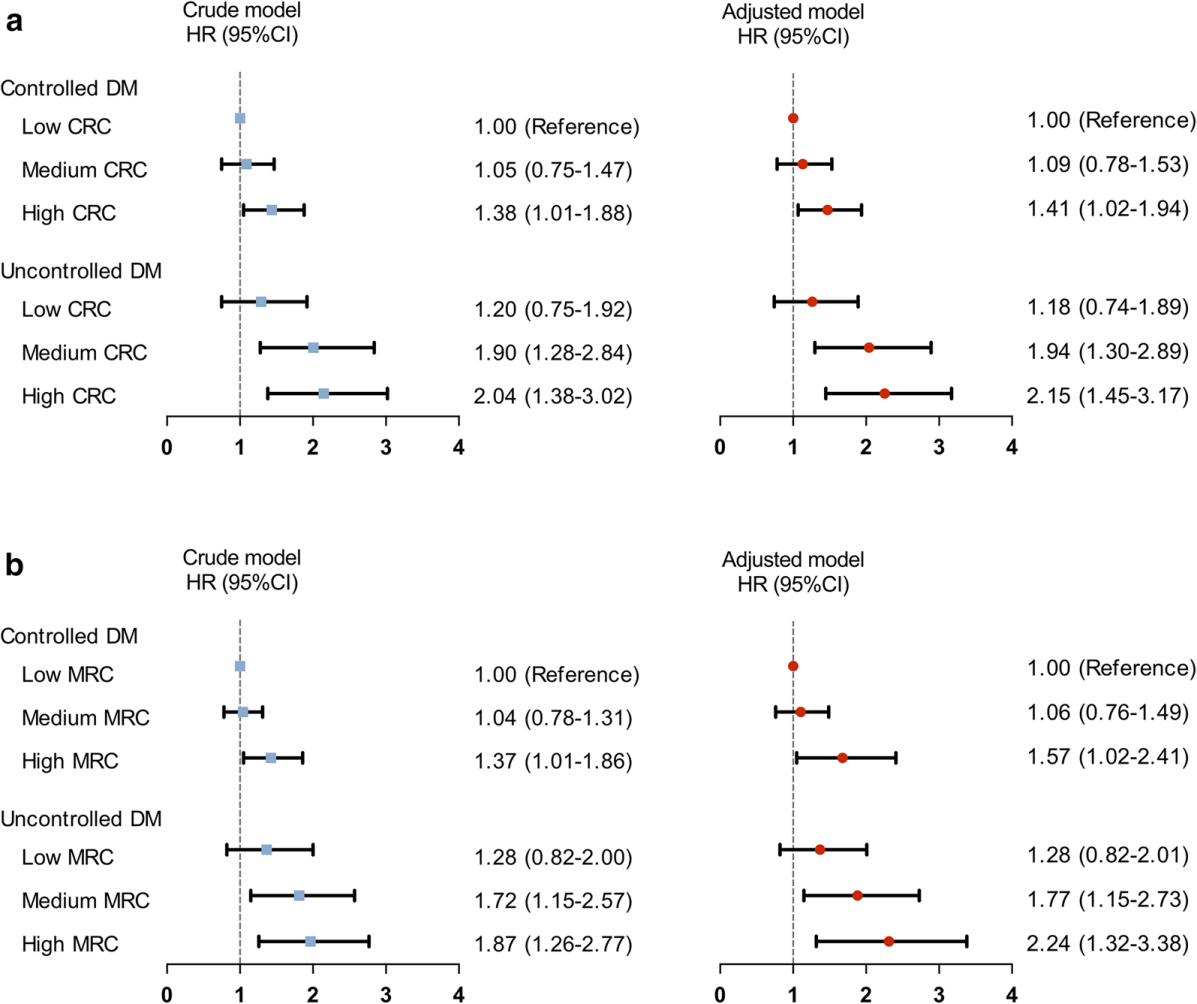
Remnant cholesterol levels in relation to cardiovascular events in patients with uncontrolled or controlled diabetes mellitus. HR, hazard ratio; CI, confidence interval; CRC, calculated remnant cholesterol; MRC, measured remnant cholesterol; NG, normoglycemia; DM, diabetes mellitus. Model adjusted for age, sex, body mass index, smoking, hypertension, baseline statin, family history of coronary artery disease, total cholesterol, low-density lipoprotein cholesterol, non-high lipoprotein cholesterol, apolipoprotein B and triglyceride

As shown in Fig. 3, when DM patients were categorized into 6 groups according to both levels of HbA1c (controlled DM defined as HbA1c < 7%) and CRC levels, those in controlled DM plus high CRC, uncontrolled DM plus medium CRC, and uncontrolled DM plus high CRC had 1.41-fold, 1.94- fold and 2.15-fold higher risk of MACEs (HR: 1.41, 95% CI 1.02–1.94; HR: 1.94, 95% CI 1.30–2.89; HR: 2.15, 95% CI 1.45–3.17, respectively). Similar results were found when both HbA1c levels and MRC status were incorporated as stratifying factors.

## Discussion

Patients with DM or pre-DM have been reported to have elevated plasma RC levels and high risk for developing CAD [19, 20]. Therefore, these populations represent a special cohort that deserve to pay more attention for the prevention of CAD [15]. In this multi-center prospective study with 5-year follow-up, the associations between CRC, MRC, and MACEs were investigated in 4331 angiography-proven CAD patients with different glucose metabolism status. The major findings were that high CRC and MRC levels as categorical and continuous variables were independent risk factors for MACEs. Interestingly, when patients were simply divided into the three groups by glucose metabolism status, Cox regression analysis showed that patients with DM but not those with pre-DM had a higher risk of MACEs. When patients were categorized according to both the status of glucose metabolism and RC levels, patients in pre-DM plus the highest tertiles of CRC and MRC had 1.64- and 1.98- fold increased risk of MACEs compared with that in patients in NG and lowest RC tertiles. Moreover, high RC levels were positively associated with MACEs in patients with uncontrolled DM. Taken together, this is the first study to show that elevated levels of plasma RC are independent prognostic factors for patients with CAD and pre-DM, which might provide new information on the necessity of monitoring RC in patients with impaired glucose metabolism for CAD risk assessment.

Elevated LDL-C is a well-known risk factor for CAD, which is commonly considered as the primary therapy target [3, 4]. However, after reduction of LDL-C to recommended levels, there is still a considerable residual risk of MACEs [5]. A growing amount of studies supported the notion that RC might contribute to this residual risk, which is of particular interest based on the fact that burgeoning prevalence of DM is associated with increased TG levels and its potential intersection with CAD [6, 7]. Emerging evidence indicated that RC was capable of converging a variety of proatherogenic effects, including monocyte activation, upregulation of proinflammatory cytokines, and increased prothrombotic factors production [5, 8]. In-vitro and animal investigations provided the evidence that elevated RC levels could lead to atherosclerosis in the same way as elevated levels of LDL-C by penetrating the arterial wall, being taken up by macrophage and causing foam cell formation. These data may suggest that RC is more important than TG to explain the residual risk though their circulating concentrations are correlated [24–26]. Numerous clinical studies also indicated that high RC concentrations were related to increased risk for atherosclerosis and CAD [27]. A recent study showed that RC was associated with coronary atheroma progression independent of conventional lipid parameters [28]. In addition to observational studies, a number of genetic studies have strongly shown that higher RC is a causal risk factor for CAD [2, 9]. More recently, the latest guideline for dyslipidemia management underlined the atherogenic effect of apoB and revealed that the clinical benefit of lipid-lowering therapy might attribute to the reduction of apoB-containing particles, which mostly referred to RC [3]. Hence, the atherogenic effects of RC may explain the associations with an increased incidence of MACEs, as demonstrated in the present study.

Our primary finding of the association between RC levels and MACEs is an extension of previous cross-sectional studies including ours [29, 30]. Several prior studies on the secondary prevention of CAD have detected an association of high RC levels with increased risk for cardiovascular outcomes, whereas others have found no such correlation. Martin et al. [31] published a report in which plasma RC was examined in 2465 American patients with MI and demonstrated that higher RC levels were associated with lower 2-year all-cause mortality after adjustment for multiple risk factors. However, a prospective study enrolled 135 patients with CAD and found that fasting RC independently predicted the development of events during a median follow-up of 2.2 years [32]. In Copenhagen Ischemic Heart Disease Study, increased non-fasting RC levels assessed in 5414 Danish patients with ischemic heart disease were related to all-cause mortality [12]. In our study, we investigated the relations of CRC and MRC levels to MACEs in patients with CAD. Importantly, coincided with previous studies, we found that patients with elevated CRC and MRC, especially those in the upper tertiles, were at high risk for MACEs after adjustment for traditional cardiovascular risk factors including statin use or TG at 5-year follow-up. Of note, although TG was associated with MACEs in the univariate COX analysis, it lost predictability in the multivariate Cox analysis. This result was in accordance with previous studies that RC levels were independent predictors for MACEs irrespective of LDL-C and TG levels [10, 33, 34]. Besides, our findings added to the evidence concerning RC and MACEs from Caucasian populations to Chinese populations. What’s more, we firstly integrated different forms of RC and compared their prognostic values in one cohort study. Thus, our study provided additional information that measuring plasma RC levels might be clinically relevant in secondary prevention to identify patients at risk of MACEs.

Another important finding of our present study was that elevated levels of RC consistently presented as prognostic indictors under different glucose metabolism status. Previous studies have provided stable evidence that CAD was a common comorbidity and the leading cause of death in patients with DM and pre-DM [13, 14]. Interestingly, high plasma RC was overproduced in insulin-resistant state and might play a crucial role in the pathogenesis of CAD in pre-DM or DM [15]. Consequently, evaluating the combination effect of high RC and DM or pre-DM status may provide new insight into the cardiovascular events and metabolic risk estimation. In a case–control study with 240 MI patients and 1.7-year follow-up, Fukushima et al. [10] showed that increased levels of RC were positively associated with future coronary events in patients with CAD and DM. Our previous study enrolling 238 patients CAD and DM showed a positive but non-statistically significant association between CRC and MACEs during one-year follow-up [27]. The current study provided strong evidence about the prognostic value of RC in patients with DM based on a large-scale cohort study with long-term follow-up. Although these two previous studies with small sample size reported the association between RC and MACEs in DM, no study regarding the joint effect of high RC and pre-DM on the risk of MACEs is currently available. In the present study, we not only examined the prognosis of RC in patients with CAD but also gave special attention to the impacts of high RC plus different glucose metabolic status on cardiovascular outcomes. Hence, our study was the first to report that patients with pre-DM companied by high CRC and MRC had 1.64-, and 1.98-fold increased risk of MACEs, respectively, indicating the clinical importance of RC measurement and intervention in patients with impaired glucose metabolism.

Of note, there is no uniform definition of RC currently and several methods for RC measurement have been used due to the heterogeneous nature of macromolecules [6, 35]. The plasma RC levels were remarkably different across studies in which ultracentrifugation, agarose gel electrophoresis, and immunoseparation were separately used, indicating their sensitivities were significantly different [10, 12, 15, 27]. In the present study, RC was measured by a fully automated detergent-based and time-saving homogenous assay confirmed by pervious high-quality studies [10, 12]. Moreover, the present study showed that CRC and MRC had similar credibility of MACE risk. In view of the convenient and less time-consuming character, our data suggested that directly calculated RC might be sufficient for prognostication and therapeutic decision-making in real-world clinic practice.

Although non-HDL-C and apoB are usually recommended as surrogate measures for RC, non-statistically significant HRs for the associations of non-HDL-C and apoB with MACEs were obtained in the current study. Considering the mean RC levels were far less than non-HDL-C in this study, the atherogenicity of RC might give a limited contribution to that of non-HDL-C [36]. In fact, the accurate measurement of each atherogenic cholesterol fraction (LDL-C vs. RC) is important to determine their relative contribution since the advent of novel lipid-lowering drugs and we move towards more personalized medicine. A post hoc analysis of TNT trial showed that the intensive lipid-lowering therapy among those with higher RC was of benefit for cardiovascular risk reduction [11]. Liraglutide, icosapent ethyl and peroxisome proliferator-activated receptor alpha modulators are novel candidates for reducing RC [37–39]. Phase I clinical trial antisense inhibition of apolipoprotein C-III showed decreased TG levels and Phase III studies are anticipated [40]. Interfering RNAs and monoclonal antibodies of apoC-III are also reported to reduce TG and TRL-C levels in the circulation [41]. Taken together, these data indicate that RC may be both a prognostic marker and a potential target for future therapeutic intervention.

The present study had several limitations. Firstly, circulating RC was measured once at baseline and the on-treatment RC may be more clinically relevant. Secondly, this was a study only enrolled Chinese patients with CAD. Whether our results could be generalized to other populations need further investigation. Finally, despite adjustments for potential known confounding variables in multivariable Cox regression analysis, we cannot exclude a possible residual bias because of that we did not assess the all metabolic factors and parameters about insulin resistance due to the features of patients in our study.

## Conclusion

In conclusion, in this multi-center prospective study, for the first time, we found that the pre-DM patients who had high RC tended to present worse prognosis when presented as calculated or directly measured forms. Moreover, we also demonstrated that high levels of RC were significant predictors of MACEs in patients having both CAD and DM independent of traditional risk factors, suggesting that assessing RC levels in CAD patients with DM or pre-DM might be likely to have clinical utility in terms of cardiovascular risk stratification and future intervention.

## Supplementary information

**Additional file 1: Table S1.** Correlations of lipoproteins and lipids. **Table S2.** Cox proportional hazards regression analysis of the CRC with events. **Table S3.** Cox proportional hazards regression analysis of the MRC with events. **Table S4.** Cox regression analysis according to different RC levels or glucose metabolism. **Table S5.** Cox regression analysis according to RC levels in different glucose metabolism status as continuous variables. **Figure S1.** Flowchart of the study. **Figure S2.** Association of calculated RC and directly measured RC levels. **Figure S3.** Age- and sex- adjusted restricted cubic spline plot of RC and risk of cardiovascular events. **Figure S4.** Kaplan–Meier analysis according to different glucose metabolism status.

## Data Availability

The datasets used and analyzed during the current study are available from the corresponding author on reasonable request.

## Abbreviations

RC: Remnant cholesterol
TRL: Triglyceride-rich lipoprotein
CAD: Coronary artery disease
DM: Diabetes mellitus
MACE: Major adverse cardiovascular event
HR: Hazard ratio
HbA1c: Hemoglobin A1c
