# Supplementary material for: The longitudinal association of remnant cholesterol with cardiovascular outcomes in patients with diabetes and pre-diabetes
Source: Cardiovasc Diabetol. 2020 Jul 6;19:104. doi: 10.1186/s12933-020-01076-7 (PMC7339517; doi:10.1186/s12933-020-01076-7)

Supplementary Online Content

Supplementary Methods

Measurement of RC

RC is the cholesterol content of triglyceride-rich lipoproteins (TRLs), and also called TRL-cholesterol (TRL-C). The TRL-C test kit enables a fully automated assay for quantification of TRL-C. The test is completed within 10 minutes. The assay consists of two-step reactions. In the first step, non-TRL-lipoproteins are decomposed by a surfactant, cholesterol esterase (CHE) with a molecular weight above 40 kDa and cholesterol oxidase (CO). The cholesterol released from these lipoprotein particles is then degraded finally to water and oxygen through the hydrolysis by the CHE, oxidization by the CO, and subsequent reaction of catalase against intermediately produced hydrogen peroxides during the reaction.

In the second step, another type of surfactant reacts with TRL. The catalase in the reaction mixture is inhibited by sodium azide. The hydrogen peroxides produced from the reaction of the released cholesterol with the CHE (molecular weight below 40 kDa) and CO, develop a purple-red color with the coupler in the presence of peroxidase (POD). Adapted from material provided by Denka Seiken.

1st step: Degradation of non-TRL cholesterol

$$\text{non-TRL-lipoproteins} \xrightarrow{\text{CHE \& CO}} \text{Cholestenone} + \text{Fatty acid} + H_2O_2$$

$$2H_2O_2 \xrightarrow{\text{catalase}} 2H_2O + O_2$$

2nd step: Determination of TRL cholesterol

$$\text{TRL} \xrightarrow{\text{CHE \& CO}} \text{Cholestenone} + \text{Fatty acid} + H_2O_2$$

$$2\ H_2O_2 + \text{4-aminoantipyrine} + \text{TOOS*} \xrightarrow{\text{POD}} \text{Purple-red color} + 4\ H_2O$$

* N-Ethyl-N- (2-hydroxy-3-sulfopropyl)-3-methylaniline

Table S1. Correlations of lipoproteins and lipids.

Variables	CRC		MRC	
	**r**	**p-value**	**r**	**p-value**
TC	0.369	0.001	0.538	0.001
LDL-C	0.165	0.001	0.466	0.001
HDL-C	-0.252	0.001	-0.152	0.001
TG	0.792	0.001	0.789	0.001
Lp(a)	0.01	0.976	0.019	0.221
ApoAI	-0.035	0.023	0.039	0.01
ApoB	0.337	0.001	0.494	0.001
BMI	0.129	0.001	0.159	0.001
Glucose	0.057	0.001	0.094	0.001
HbA1C	0.081	0.001	0.06	0.001
HsCRP	0.061	0.001	0.055	0.001

Table S2. Cox proportional hazards regression analysis of the CRC with events.

Variables	Univariate Model		Multivariate Model	
	**HR(95%CI)**	**p-value**	**HR(95%CI)**	**p-value**
Age	1.00 (1.00-1.01)	0.066	1.00 (1.00-1.01)	0.038
Sex	1.17 (0.98-1.40)	0.09	-	
BMI	1.00 (0.97-1.03)	0.951	-	
Hypertension	0.84 (0.70-1.01)	0.067	-	
Diabetes	1.05 (0.89-1.24)	0.580	-	
Smoking	0.91 (0.77-1.07)	0.248	-	
Family history of CHD	1.18 (0.92-1.53)	0.188	-	
Baseline Statin	0.89 (0.75-1.06)	0.198	-	
TC	1.08 (0.99-1.16)	0.068	-	
non-HDL-C	1.08 (0.99-1.17)	0.064	-	
ApoB	1.23 (0.94-1.62)	0.130	-	
HDL-C	0.87 (0.65-1.16)	0.338	-	
LDL-C	1.06 (0.96-1.16)	0.238	-	
TG	1.17 (1.04-1.30)	0.004	-	
Log-CRC	1.34 (1.15-1.57)	<0.001	1.97 (1.28-3.02)	0.002

Table S3. Cox proportional hazards regression analysis of the MRC with events.

Variables	Univariate Model		Multivariate Model	
	**HR(95%CI)**	**p-value**	**HR(95%CI)**	**p-value**
Age	1.00 (1.00-1.01)	0.066	1.00 (1.00-1.01)	0.027
Sex	1.17 (0.98-1.40)	0.09	-	
BMI	1.00 (0.97-1.03)	0.951	-	
Hypertension	0.84 (0.70-1.01)	0.067	-	
Diabetes	1.05 (0.89-1.24)	0.580	-	
Smoking	0.91 (0.77-1.07)	0.248	-	
Family history of CHD	1.18 (0.92-1.53)	0.188	-	
Baseline Statin	0.89 (0.75-1.06)	0.198	-	
TC	1.08 (0.99-1.16)	0.068	-	
non-HDL-C	1.08 (0.99-1.17)	0.064	-	
ApoB	1.23 (0.94-1.62)	0.130	-	
HDL-C	0.87 (0.65-1.16)	0.238	-	
LDL-C	1.06 (0.96-1.16)	0.238	-	
TG	1.17 (1.04-1.30)	0.004	-	
Log-MRC	1.52 (1.26-1.84)	<0.001	1.54 (1.27-1.86)	<0.001

Table S4. Cox regression analysis according to different RC levels or glucose metabolism.

Variables	Events/Subjects	Univariate Model		Multivariate Model	
		HR(95%CI)	p-value	HR(95%CI)	p-value
CRC					
Tertile 1	151/1443	1.00 (Reference)	-	1.00 (Reference)	-
Tertile 2	169/1444	1.09 (0.88-1.36)	0.441	1.10 (0.88-1.36)	0.418
Tertile 3	221/1444	1.45 (1.18-1.79)	0.001	1.47 (1.20-1.81)	0.001
MRC					
Tertile 1	155/1443	1.00 (Reference)	-	1.00 (Reference)	-
Tertile 2	171/1444	1.10 (0.89-1.37)	0.372	1.10 (0.89-1.37)	0.383
Tertile 3	215/1444	1.41 (1.15-1.73)	0.001	1.42 (1.16-1.75)	0.001
Diabetic status					
NGR	78/776	1.00 (Reference)	-	1.00 (Reference)	-
Pre-DM	136/1163	1.17 (0.89-1.55)	0.269	1.14 (0.86-1.50)	0.374
DM	327/2392	1.40 (1.09-1.79)	0.008	1.35 (1.06-1.73)	0.017

Table S5. Cox regression analysis according to RC levels in different glucose metabolism status as continuous variables.

Variables	HR(95%CI)			
	Crude Model	p-value	Adjusted Model	p-value
Log-CRC				
NG	1.09 (0.75-1.59)	0.655	1.02 (1.00-1.04)	0.107
Pre-DM	1.58 (1.15-2.17)	0.004	1.59 (1.16-2.18)	0.004
DM	1.31 (1.07-1.59)	0.008	1.40 (1.04-1.88)	0.029
Log-MRC				
NG	1.94 (1.19-3.19)	0.009	1.96 (1.20-3.20)	0.008
Pre-DM	1.71 (1.17-2.51)	0.006	1.80 (1.16-2.78)	0.009
DM	1.32 (1.04-1.69)	0.024	1.38 (1.08-1.76)	0.011

Figure S1. Flowchart of the study.

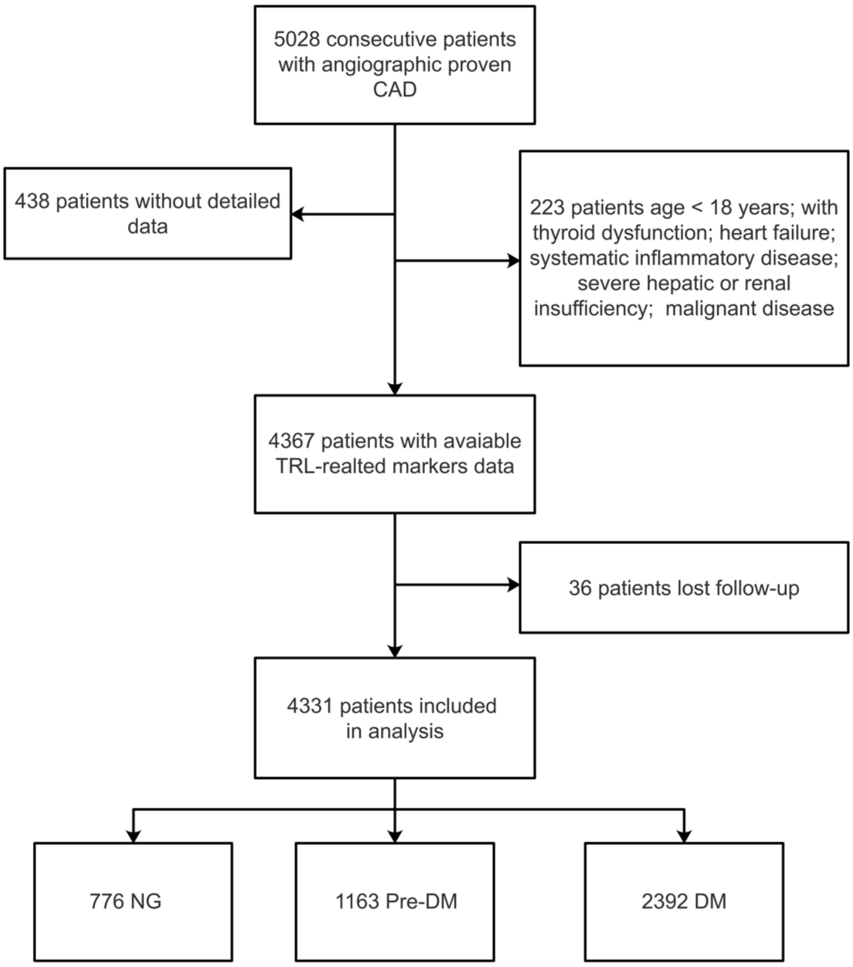

Figure S2. Association of calculated RC and directly measured RC levels.

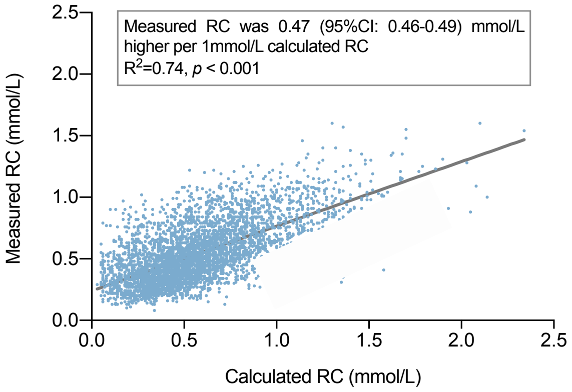

Figure S3. Age- and sex- adjusted restricted cubic spline plot of RC and risk of cardiovascular events.

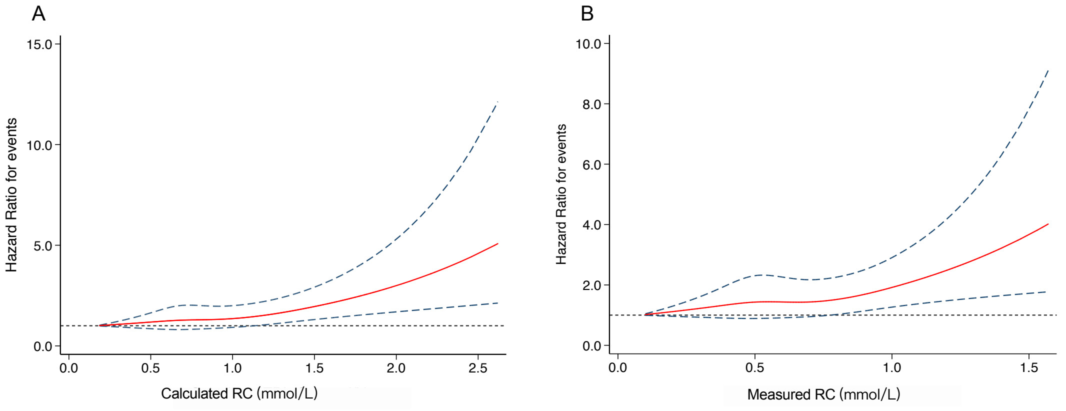

Figure S4. Kaplan-Meier analysis according to different glucose metabolism status.

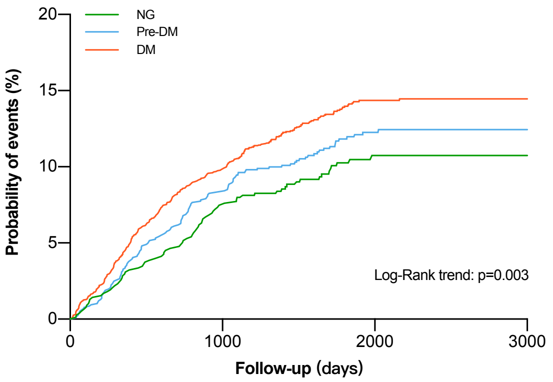

Supplement: Supplementary file 1 — Additional file 1: Table S1. Correlations of lipoproteins and lipids. Table S2. Cox proportional hazards regression analysis of the CRC with events. Table S3. Cox proportional hazards regression analysis of the MRC with events. Table S4. Cox regression analysis according to different RC levels or glucose metabolism. Table S5. Cox regression analysis according to RC levels in different glucose metabolism status as continuous variables. Figure S1. Flowchart of the study. Figure S2. Association of calculated RC and directly measured RC levels. Figure S3. Age- and sex- adjusted restricted cubic spline plot of RC and risk of cardiovascular events. Figure S4. Kaplan–Meier analysis according to different glucose metabolism status. [file 12933_2020_1076_MOESM1_ESM.pdf]
